# Tumor clusters with divergent inflammation and human retroelement expression determine the clinical outcome of patients with serous ovarian cancer

**DOI:** 10.1002/1878-0261.70067

**Published:** 2025-06-10

**Authors:** Laura Glossner, Markus Eckstein, Christoph Mark, Matthias W. Beckmann, Arndt Hartmann, Pamela L. Strissel, Reiner Strick

**Affiliations:** ^1^ University Hospital Erlangen, Institute of Pathology, Comprehensive Cancer Center Erlangen‐EMN (CCC ER‐EMN), Erlangen Friedrich‐Alexander University Erlangen‐Nürnberg Germany; ^2^ Department of Physics Friedrich‐Alexander University Erlangen‐Nürnberg Germany; ^3^ Artifact Research, GmbH Nürnberg Germany; ^4^ Department of Gynecology and Obstetrics, Laboratory for Molecular Medicine, Comprehensive Cancer Center Erlangen‐EMN (CCC ER‐EMN), Erlangen University Hospital Erlangen Germany; ^5^ Adjunct Affiliation With Department of Radiation Oncology University of Maryland School of Medicine Baltimore MD USA

**Keywords:** dsRNA, ERV, inflammation, ovarian carcinoma, tumor immune microenvironment, viral mimicry

## Abstract

High‐grade serous ovarian carcinoma (HGSOC) associates with the worst patient outcome. Understanding the tumor environment in terms of quantifying endogenous retroviruses (*ERVs*) and *LINE‐1* expression and their correlations with inflammation genes, checkpoint inhibitors and patient survival is needed. Analysis of 102 treatment‐naïve HGSOC and control tissues for *ERVs*, *LINE‐1*, inflammation and immune checkpoints identified five clusters with diverse patient recurrence‐free survivals. One cluster termed Triple‐I with the best patient survival showed the highest number of tumor infiltrating lymphocytes along with 22 overexpressed genes, including *CXCL9* and *AIM2*. However, Triple‐I associated with the lowest *ERV/LINE‐1* expression. The tumor cluster with the second‐best patient survival had both high *ERV/LINE‐1* expression and inflammation. Multiplex‐immunohistochemistry revealed CD28 protein solely on immune cells, without co‐expression of the inhibitory CTLA4 receptor. The largest tumor cluster with high *ERV/LINE‐1* expression but low inflammation showed a significant low gene expression of the dsRNA sensors *MDA5* and *RIG‐I* supporting an aberrant block in IFN signaling. Our study represents an intrinsic ‘molecular and immunological snapshot’ of the HGSOC tumor environment important for understanding retroelements and inflammation for clinical relevance.

## Graphical abstract

Analysis of treatment‐naïve high‐grade serous ovarian carcinoma (HGSOC) and control tissues for ERVs, LINE‐1 (L1), inflammation, and immune checkpoints identified five clusters with diverse patient recurrence‐free survivals. An inflammation score was calculated and correlated with retroelement expression, where one novel cluster (Triple‐I) with high inflammation and the lowest retroelement expression (no viral mimicry) showed the best survival.

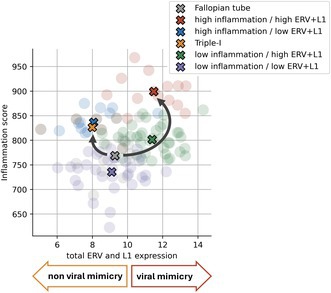

**Abbreviations**
AIM2: absent in melanoma
CXCL9: CXC motif chemokine ligand
DEG: differentially expressed gene
env: envelope
ERV: endogenous retrovirus
HGSOC: high‐grade serous ovarian carcinoma
INF: inflammation
IRGS: inflammation and regulatory gene signature
L1‐5′UTR: LINE‐1 5′ untranslated region
L1‐ORF2: LINE‐1 open reading frame 2
LINE‐1: long interspersed nuclear element‐1
RFS: recurrence‐free survivals
sTILs: stromal tumor infiltrating lymphocytes
TIME: tumor immune microenvironment

## 1 Introduction

Low or high inflammation states of tumors dictate patient survival [1]. Inflammation stems from activation and recruitment of the innate and adaptive immune responses. Antigen presentation, along with immune checkpoint receptor and ligand interaction of immune cells, is a normal part of the immune response or can be aberrant due to an adaptive response of the tumor immune microenvironment (TIME). After induction of the innate immune response and interferon (IFN) type I signaling, a possible cross talk with the adaptive immune response can occur [2]. For example, following activation of innate toll‐like receptors (TLRs), NOD‐like receptors (NLR), RIG‐I‐like receptors (RLR) and IFN‐stimulated genes like IFI16, an AIM2‐like receptor, along with cytokines (e.g., IL18) IFNG expression becomes induced. IFNG is secreted by immune cells like natural killer (NK), lymphoid, T helper, and cytotoxic T cells [3]. Along with direct cytotoxic T‐cell killing of virally infected or tumor cells, IFN signaling ultimately results in apoptosis or pyroptosis via inflammasomes [4].

Several origins of inflammation exist. For example, ‘sterile inflammation’ occurs in the absence of microorganisms and is due to endogenous signals like heat‐shock proteins, RNA or DNA recognized by TLRs and other sensors [5]. One important source of RNA that induces tumor inflammation is endogenous retroviruses (*ERVs*) [6, 7]. *ERVs* are part of the family of long terminal repeat (LTR)‐retroelements that integrated throughout the human genome from past infections [8]. *ERVs* are repetitive elements and represent 5–8% of the genome, though their total numbers vary due to mosaic forms [9]. *ERVs* are classified into class I (gamma/epsilon), class II (alpha/beta) retroviruses, and class III or spumaviruses. Several groups of *ERV* families exist, where *ERV‐K* is the most represented [10]. Although the majority of *ERV* elements are defective due to mutations and deletions over time, many *ERV* elements retained protein functionality, esp. envelope (env) proteins [11] (Fig. S1A). In general, *ERVs* are silenced in somatic tissues due to epigenetic regulation. However, *ERV* expression becomes de‐repressed during development [12] and in various tumors [13, 14] as well as in tumor staging [15]. Importantly, the role of ERV proteins as tumor antigens, with tumor‐promoting functions, may represent new therapeutic targets, which are under investigation [16, 17]. Tumor *ERV* element activation occurring intrinsically or following epigenetic inhibitor treatment, defined as viral mimicry, results in *ERV* double‐strand (ds)RNA complexes and induces IFN type I signaling via innate immunity [6, 7, 18].

Long interspersed nuclear elements (*LINE*)*‐1 (L1)* are non‐LTR retrotransposons and have accumulated mutations and deletions throughout evolution, resulting esp. in 5′UTR truncations (for review [19]). *L1* represents over half a million defective repetitive elements throughout the human genome. These defective *L1* repetitive elements can be transcribed and participate in RNA‐mediated regulatory control of cellular behaviors. In contrast, only ~ 100 active full‐length *L1* elements exist, which code for proteins involved in active retrotransposition [20]. A full‐length *L1* consists of a 5′ untranslated region (UTR), which harbors the promoter for transcription and an antisense transcriptional initiation signal for open reading frame (ORF)0, as well as two open reading frames (*ORF*)‐1 and *ORF2*, and a 3′UTR (Fig. S1A). Importantly, using imaging and biochemical analyses demonstrated that the *L1*‐*ORF2* reverse transcriptase protein preferentially binds RNA substrates and robustly produces cytosolic RNA:DNA hybrids [21, 22]. Accumulation of cellular RNA:DNA hybrids led to the activation of innate immune signaling via cGAS/STING and IFN production. On the contrary, a known active *L1* subset, *L1PA1* belonging to the *L1Hs* subfamily, and *L1PA2* are a source of dsRNA, which induces IFN‐stimulated genes (ISGs) via JAK/STAT signaling [23]. Despite that both *ERVs* and *L1* ignite IFN signaling, further research is needed to quantify high and low intrinsic tumor *ERV* and *L1* gene expression levels and how they correlate with each other, as well as with inflammation genes, checkpoint inhibitors, and patient survival. Due to the highly repetitive nature of *ERVs* and especially *LTRs*, it still remains a challenge to identify and accurately localize them within the genome using standard RNA‐seq protocols, like the *ERV‐K* family, which includes over 550 proviruses and over 6400 *LTRs* [24]. For example, when using RNA‐seq, multiple mapping reads can result in elimination of abundant *ERVs* or even miss individual *ERV* elements and their locations when using empirical models. Implementing standard short 76–150 nucleotide RNA‐seq reads for repetitive regions can also result in multiple matches and thus localize *ERVs* to erroneous locations yielding inaccurate up‐ or downregulations [25, 26]. Nonetheless, different patient tumor samples analyzed for *ERVs* using RNA‐seq technologies identified common *ERV* subfamilies, for example, solo *LTRs*, *MER*, *ERV‐E*, and *ERV‐H* with an emphasis on inflammation [27, 28, 29].

High‐grade serous ovarian carcinoma (HGSOC) is the most common and lethal ovarian cancer with an overall poor patient survival between 2.8 and 5.1 years depending upon tumor infiltrating lymphocytes (TILs). HGSOC has been hypothesized to originate from the fallopian tube or ovarian surface epithelium [30, 31, 32]. Treatment of HGSOC continues to be difficult and so far immunotherapy has fallen short of expectations [33], despite the existence of CD8^+^ T‐cell infiltration [34]. More research is needed to understand checkpoint regulatory molecules and their biology in the HGSOC TIME. In the present study, we quantified intrinsic expression levels of multiple codogenic and noncodogenic *ERV* families, the *L1‐5′UTR* and the *L1‐ORF2* in 102 HGSOC of patients without prior treatment. Our methodology implemented specific gene primers and absolute qPCR, where expression levels were fully quantified using cloned genes. *ERVs*, *L1‐5′UTR* and the *L1‐ORF2* expression correlated significantly with each other in HGSOC and with a gene linked with hypomethylated DNA in cancers. *ERVs* and *L1‐ORF2* gene expression also correlated significantly with tumor inflammatory genes, immune cells, immune checkpoint regulatory genes and patient survival. We identified landmark genes important for survival as well as determining their regulation using HGSOC cell lines. Our study represents a unique intrinsic ‘molecular snapshot’ of the HGSOC TIME before patient treatment in order to gain insight of retroelements and inflammation for clinical relevance. We present novel molecular findings regarding the biology of the TIME important for patient survival.

## 2 Materials and methods

### 2.1 Patient clinical and pathological data, tumors, and control tissues

All primary human tissue sampling and research of archived primary banked formalin‐fixed paraffin‐embedded (FFPE) tumor blocks was approved by the Ethics Committee of the Friedrich‐Alexander University Erlangen‐Nuremberg, Germany (#199_19Bc and #252_20B) in accordance with the World Medical Association Declaration of Helsinki. The experiments were undertaken with the understanding and written consent of each subject. All patient clinical and pathological data were collected from the Comprehensive Cancer Center (CCC), Erlangen (Table 1).

**Table 1:** Clinical and pathological parameters and inflammation states of the *ERV‐L1‐ORF2* tumor clusters from patients with high‐grade serous ovarian carcinoma (HGSOC). From top to the bottom: HGSOC cohort (*n* = 102), with a mean age of 61 years. All patients were Europeans where 96 of the patients were German and six patients with Polish, Turkish and Serbian Nationalities. All 102 tumors were pathologically graded as high‐grade G3 with tumor stages pT: pT1‐3; lymph node negative (pN0) or positive pN1; R = residual tumor, R0 = resection for a curative intention, R+ = residual tumor present; and from three patients, no information was available. Regarding adjuvant (adj.) chemotherapy (CT): 74 Patients were enrolled into a standard therapy treatment program with: carboplatin and paclitaxel (*n* = 62); carboplatin, paclitaxel, and bevacizumab (*n* = 2); carboplatin, paclitaxel, and gemcitabine (*n* = 1); carboplatin (*n* = 2); seven patients without available treatment information; and 28 patients received no chemotherapy treatment; tumor stroma appearance; sTILs = stromal T infiltrating lymphocytes; *ERV‐L1* expression (molecules·ng^−1^ RNA) for all tumors as high or low determined by absolute qPCR; five tumor clusters were determined by *ERV‐L1‐ORF2* total molecules·ng^−1^ RNA compared with 99 tumor inflammation genes defined as the Inflammation regulatory gene signature (IRGS). In addition, recurrence‐free survival up to 66 months (5.5 years) was available for all 102 HGSOC patients. ERV, endogenous retroviruses.

HGSOC (*n* =)	102				
Demography: European (*n* =)	102				
Grading: G3 (*n* =)	102				
pT	pT1	pT2	pT3		
*n* =	12	9	81		
pN	pN0	pN1			
*n* =	44	58			
R	R0	R+			
*n* =	78	21			
Adj. CT	No	Yes			
*n* =	28	74			
Tumor stroma Appearance	Fibrous	Myxoid	Chondromyxoid	Leiomyomatous	
*n* =	41	26	20	15	
sTILs (%) categories	1 %	2‐3 %	5 ‐10 %	15 ‐ 25 %	30 – 60 %
*n* =	36	20	25	13	8
*ERV/L1‐ORF2* expression	High	Low			
*n* =	52	50			
*ERV/L1‐ORF2/*inflammation	INFhi/*ERVhi*	INFlow/*ERVhi*	INFlow/*ERVlow*	INFhi/*ERVlow*	Triple‐I
*n* (%)	14 (13.7%)	38 (37.2%)	33 (32.3%)	11 (10.8%)	6 (5.9%)

Hematoxylin and eosin (H&E) stained tissues from archived primary FFPE tumor blocks (*n* = 102) were screened by two Pathologists (ME, AH) to confirm HGSOC histology, grading (all G3) and a tumor content of > 85%. Patients with distant metastasis were not included in this study. The mean age of the patients was 61 years (Table 1). All HGSOC H&E tissues were scored for stroma type and stromal TILs (sTILs) (Table 1). FFPE healthy nonpatient matched fallopian tubes (*n* = 13) were obtained from women undergoing elective sterilization. Healthy ovarian tissues (*n* = 16; mean age = 57 year) as partly previously described [6], were dissected from patients with HGSOC (patient matched (*n* = 2) and nonpatient matched (*n* = 3)), women undergoing surgeries for ovarian cysts (*n* = 7), dermoid (*n* = 1) or leiomyoma (*n* = 1) as well as from patients with no pathological conditions (*n* = 2).

### 2.2 RNA isolation for gene expression and direct multiplex RNA hybridization (NanoString)

All protocols were according to Kohler et al. [35]. For tumor and healthy tissues, 10 μm thick tissue cuts from two different tumor blocks per patient were pooled. Tissue cuts were treated with methylene blue, and the RNA was isolated using the Maxwell RSC RNA FFPE kit (Promega, #AS1440, Walldorf, Germany). Following DNase I treatment, the RNA was further purified and quantified. A NanoString PlexSet with 260 genes was designed from our previous studies and modified [35, 36] (Table S1A). These 260 genes included known reference genes as well as genes important for cell cycle progression, extracellular matrix (ECM) remodeling, epithelial to mesenchymal transition, tumor phenotyping, immune cell phenotypes, and inflammation. The 37 common reference genes chosen from the literature were analyzed with tumor and healthy tissue RNAs using NanoString and qPCR in order to determine the most suited reference genes [35]. According to the coefficient of variation (COV), two genes, beta‐actin (*ACTB*; HGNC:132) and Tyrosine 3‐monooxygenase/tryptophan 5‐monooxygenase activation protein zeta (*YWHAZ*; HGNC:12855) were the most constant in their expression for all HGSOC and normal fallopian tubes (Fig. S1B). Following normalization of tumor and healthy tissue RNAs with *ACTB* and *YWHAZ*, we then performed a gene‐specific analysis with 99 genes chosen from the NanoString gene panel, which correlated with the immune response (IFN pathways, nucleic acid sensors, immune cell phenotypes, and cytokines and other immune regulators). The 99 selected gene panel stated throughout the manuscript is referred to as the Inflammation Regulatory Gene Signature (IRGS) (Table S1B). For gene expression validation of NanoString and qPCR (described below), we chose one gene from the IRGS panel, *ZBP1*, for confirmation (Table S1B,C, Fig. S1C,D). Importantly, similar results were found using both gene expression techniques.

### 2.3 Quantitative and full quantitative real‐time PCR of *ERVs* , *L1‐5′UTR* and *L1‐ORF2*

All protocols for quantitative (q) and absolute qPCR using cDNA and our published *ERV* and *L1* primers (Table S1C,D) and a SYBR select master mix, were performed using a StepOne plus PCR system according to different publications (Thermo Fisher, Waltham, MA, USA) [6, 15, 17, 37]. QPCRs were normalized using the two best reference genes *ACTB* and *YWHAZ* for HGSOC and FT as described above. A fragment of the *L1‐ORF2* (660 bp) was amplified using a TF 5′ primer GAGGCCAGCATCATTCTGATACCAAAGCCG and a BR 5′ TCCTGAGACTTTGCTGAAGTTGCTTATCAGC and then cloned into a pSC‐A vector (Agilent, Waldbronn, Germany). Using primers for *L1‐ORF2* qPCR [37] (Table S1C), we calculated a slope of *y* = −3.5537 + 38.997 and *R* ^2^ of 0.9908 using a standard curve with the cloned *L1‐ORF2* fragment. In addition, the *L1‐5′UTR* was also fully quantitatively analyzed, where the *5′UTR* gene region from nucleotide 365 till 854 was chosen according to the human L1 element LRE2 from chromosome 1q (U09116.1). This *L1*‐5′UTR region was synthesized (BioCat, Heidelberg, Germany) and cloned into pBlueScript II KS(+) (Agilent Tech., USA). Using primers for *L1‐5′UTR* [38] (Table S1C), we calculated a slope of *y* = −3.129 + 39.942 and *R* ^2^ of 0.9973 using a standard curve with the cloned *L1‐5′UTR* fragment. All *ERVs*, *L1‐5′UTR* and *L1‐ORF2* qPCR expression values were transformed into molecules·ng^−1^ total RNA using a standard curve with a cycle threshold (cT) value against the log of amount of standard applying the known copy number of each cloned gene as an external standard [15]. *ERV* total represents the combined expression of all *ERVs* studied in this investigation (Table S1C,D). We also included a known gene for genomic de‐repression as a PCR control. We chose the cancer testis antigen *CCCTC‐*binding factor like (*CTCFL*, also called *BORIS*), which is hypomethylated in several cancers [39], but especially in HGSOC compared with control tissues [40]. All additional qPCR primers are described in Table S1C.

### 2.4 Immunohistochemistry (IHC) of immune cells

Nine antibodies previously described, CD4, CD8, FOXP3, LAG3, CD80, PD1, PD‐L1, CTLA4, and MHC‐I, specific for cell surface markers were hybridized to a newly constructed tissue microarray (TMA) representing all 102 HGSOC at the Institute of Pathology to identify different immune cells and immune checkpoint inhibitors or receptor protein molecules according to Pfannstiel et al. [41]. For CD28 (antibody clone RM404, Novus Biologicals, Centennial, CO, USA) a dilution of 1 : 1000 was hybridized to the above TMA and patient‐matched lymph node tissues (*n* = 14) along with the CTLA4 antibody. For Multiplex IHC, both CTLA‐4 (clone BSB‐88, from Bio SB, Medac GmbH, Wedel, Germany) and CD28 antibodies were hybridized to HGSOC tissue cuts (*n* = 9). Briefly, following tissue rehydration, samples were incubated in TRIS buffer pH 6 for 15 min; then, the CTLA4 antibody (1 : 200) was incubated overnight at 4 °C. For detection of CTLA4, the polymer alkaline phosphatase (AP) with permanent red (ZytoChem Plus (AP) Polymer Kit', POLAP‐100, from Zytomed, Berlin, Germany) was used. The CD28 antibody (1 : 500) was hybridized for 2 h at RT and then detected using the Polymer‐AP and Permanent Green (Zytomed). Lastly, all 102 HGSOC H&E stained tissues were analyzed (ME; LG) to quantify the percentage of sTILs. STILs were scored in tumor stroma according to a validated methodology of the International Working Group on TILs as described in Pfannstiel et al. [41].

### 2.5 Cell culture studies

Two HGSOC cell lines, TyKnu (RRID:CVCL_1776) and Kuramochi (RRID:CVCL_1345), were obtained following genomic and RNA expression characterization [42] as well as authentication from Professor S. Baylin, Johns Hopkins University, USA. TyKnu and Kuramochi cell lines were authenticated using 20 STR (short tandem repeat) markers and showed 100% identity with the reference cell markers according to Papp et al. [42] (see Table S1 within). All cell culture experiments were performed within 3 years after authentication. Both cell lines were maintained in RPMI 1640 media supplemented with 10% FCS, 1 × NEAA (Thermo Fisher), l‐glutamine (Thermo Fisher) at 37 °C, 5% CO_2_, and routinely analyzed for mycoplasma. All experiments with the above cell lines were performed with mycoplasma‐free cells. Cell lines were treated with IFNG (R&D Systems, Minneapolis, MN, USA) (10 and 50 ng·mL^−1^) for 21 h, and then, the RNA was extracted with TriFast (VWR, Darmstadt, Germany) according to the manufacturer's recommendations. Real‐time qPCR for *CXCL9* and *AIM2* was performed as described above (see Table S1C for primers).

### 2.6 Bioinformatic analyses

The sas jmp, SAS Inst., Heidelberg, Germany 13.2 computer software was used to generate gene expression heatmaps, Kaplan–Meier patient survival analyses, and COX regression analyses [41]. Specific protein expression represented by each IHC hybridization was quantified using qupath software according to Pfannstiel et al. [41] for analyzing tumor centers, invasion fronts, and then the mean sum calculated. The computer software qupath V0.5.0 was used along with an artificial intelligence (AI) training tool for quantification of double positive CD28^+^ (green) and CTLA4^+^ cells (red) and single signals for CD28^+^ (green) cells using Multiplex IHC. The localization of all *ERV* and *L1* primer pairs on human chromosomes was performed with Blast® (https://blast.ncbi.nlm.nih.gov/Blast.cgi). For *ERV* primer pairs, a ≥ 94% identity, 0 gaps, and a score of 38.3–44.6 bits was designated, which allows for 1 or 2 mismatches in the primer sequences, as well as for 100% identity (Table S1E). We additionally performed an *in silico* chromosome analysis of *L1‐5′UTR* and *L1‐ORF2* primer pairs with 100% identity (TF and BR) and identical amplicon length using the NCBI chromosome alignment browser: https://www.ncbi.nlm.nih.gov/gdv/browser/genome/?id=GCF_000001405.40&chr=1. The exact nucleotide localizations of each *L1‐5′UTR* and *L1‐ORF2* primer pair using GRCh38.p14 reference primary assembly with the RS_2024_08 annotation release (Table S1E) were identified (Table S1F,G).

In order to identify significantly differentially expressed genes (DEGs) between the HGSOC inflammation *ERV‐L1‐ORF2* clusters using the total NanoString or IRGS expression values, specific statistical tests, like deseq2 (default analyses pipeline requiring NanoString raw count data as input), Welch's *t*‐test to derive per gene, nonadjusted *P*‐values for log2‐fold change and the Benjamini–Hochberg procedure to adjust *P*‐values for multiple testing were used. These statistical tests resulted in a significance level set as *P* < 0.01 with a minimal difference of 2 in log2. CIBERSORT [43] identified immune cells associating with the five inflammation*/ERV‐L1‐ORF2* tumor clusters. To obtain the total abundance of different immune cell types, designated as absolute expression values based from 225 NanoString gene expression (223 genes plus *ACTB* and *YWHAZ*); values were averaged from all tumor clusters, including fallopian tubes. Normalization with respect to individual inflammation tumor clusters yielded separate immune cell populations in per cent. The abundance of immune cell types within the five inflammation tumor clusters compared with fallopian tubes were obtained by quantifying the log2‐fold change.

A pruning approach was employed to determine minimal sets of genes classifying the different five inflammation *ERV‐L1‐ORF2* tumor clusters, using the total NanoString gene set or the IRGS NanoString panel along with individual *ERV‐L1‐ORF2* gene expression levels. The pruning procedure starts by fitting a logistic regression model to all available gene expression values to predict whether each sample is part of a selected cluster (using leave‐one‐out cross‐validation). After model fitting, the importance of each gene was determined and the least important gene was pruned from the dataset. Subsequently, the logistic regression model was fitted again using the smaller set of gene expression values, and then the least important gene was removed. The minimal gene set was identified by the model fit that attains the highest precision in classifying the correct cluster (if multiple gene sets attain an identical precision, we chose the smallest one). The inflammation score was calculated for each tumor sample from the inflammation‐low and inflammation‐high clusters as the sum of the log2‐expression levels of 99 IRGS NanoString genes. The inflammation score summarizes the state of HGSOC inflammation in a single continuous metric. This score was directly compared with *ERV* total and *L1‐ORF2* expression values for each inflammation cluster.

## 3 Results

### 3.1 Expression of *ERVs* , *L1‐5′* *UTR*, and *L1‐ORF2* in HGSOC and control tissues

To quantify *ERV*, *L1‐5′UTR*, and *L1‐ORF2* gene expression in HGSOC and healthy tissues, we used absolute qPCR developed previously by us [6, 14, 15]. This methodology allows for a direct comparison of *ERV* and *L1* molecules between different tissues. Our designed *ERV* primer pairs identify all major *ERV* families in the genome with low and high numbers of groups classified according to specific *LTR* families (Table S1C,D). For example, *ERV‐Fc*, *ERV‐FRD*, *ERV‐Fb*, *ERV‐F(XA34)*, *ERV‐H*, *ERV‐W*, *ERV‐K*, *ERV‐R*, *ERV‐E*, *ERV‐Pb*, *ERV‐T*, *ERV‐Rb*, *MER34*, *HRES1*, and *ERV9*, including families with multiple groups on different chromosomes, like *ERV‐K* with 27 groups (Table S1C,D). Analyzing the human genome for an alignment of all *ERV* primer pairs with 100% or ≥ 94% identity using Blast® resulted in various localizations for each chromosome (Table S1E). For example, 11 (100% identity) or 75 (≥ 94% identity) different chromosomal loci were found for *ERV‐Kenv* primer pairs, but only one location for *ERV‐Rb* (chromosome 3) using both alignments. A chromosomal alignment of the *L1‐5′UTR* and *L1‐ORF2* primer pairs with 100% identity and an identical amplicon length yielded 1281 and 555 loci, respectively (Table S1E–G). Interestingly, these results showed a higher number of chromosomal localizations for the *L1‐5′UTR* versus *L1‐ORF2* using these specific primer sequences (Table S1F,G). Although several publications have described frequent 5′ truncations of *L1* (for review [19]), it is important to note that the higher number of *L1‐5′UTR* versus *L1‐ORF2* chromosomal localizations was identified with these specific primer pairs using 100% identity (Table S1C). Thus, additional bioinformatical genomic analyses are needed regarding *L1‐5′UTR* and *L1‐ORF2*.

**Fig. 1:**
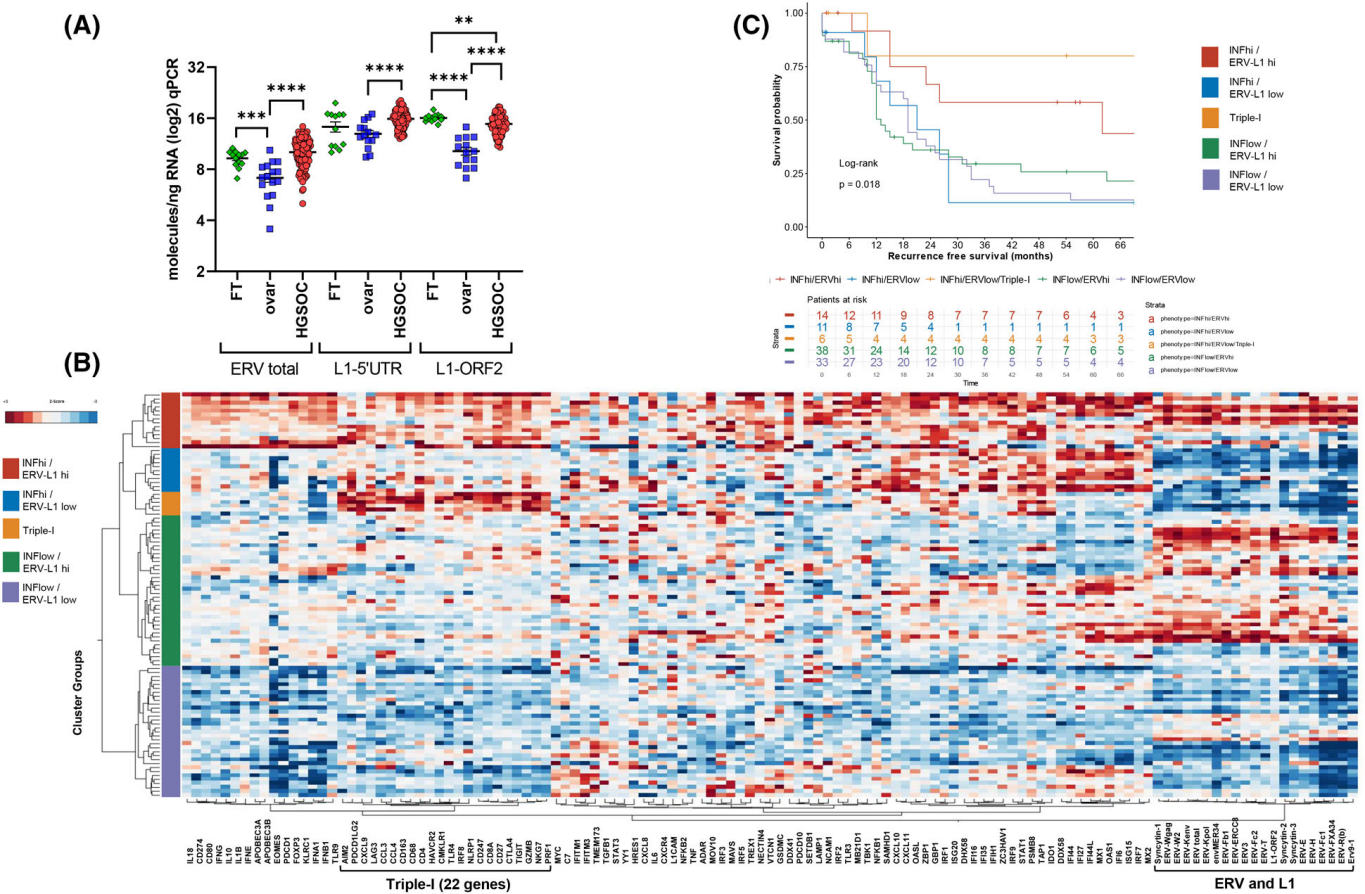
(A) High‐grade serous ovarian carcinoma (HGSOC) retroelement gene expression results of the total endogenous retroviruses (*ERVs*), *L1‐5′UTR* and *L1‐ORF2* compared with healthy fallopian tube and ovarian control tissues. Absolute quantification real‐time PCR results for each tissue show the gene expression levels (green color = Fallopian tubes (FT) (*n* = 13); blue = ovarian (ovar) (*n* = 16) and red = HGSOC (*n* = 102)). The *ERV* total represents the combined expression of all *ERVs* studied in this investigation (Table S1C,D). The total *L1‐5′UTR* and *L1‐ORF2* molecules·ng^−1^ RNA levels per tissue are also shown (Table S1C). Graph shows log2 of molecules·ng^−1^ (RNA) for all tissues. Mean values (all log2) for *ERV* total expression in fallopian tubes was 9.27, in ovar 7.2 and in HGSOC 10.1; for L1‐5′UTR in fallopian tubes 14.24, in ovar 12.94 and HGSOC 15.88; for L1‐ORF2 in fallopian tubes 16.07, in ovar 10.23 and in HGSOC 14.79. Graph bars represent SEM. The two‐tailed Mann–Whitney *U*‐test was used for *P*‐values. **P: 0.0058; ***P: 0.0007; ****P < 0.0001. (B) Heatmap of HGSOC (*n* = 102) leading to the discovery of five different significant Inflammation *ERV‐L1* clusters using jmp computer software. Gene expression of HGSOC tumor clusters identifying five significant inflammation *ERV*‐*L1* clusters in context to the 99 Interferon regulatory gene signature (IRGS) using NanoString technology. Inflammation = INF; *ERV* = endogenous retroviruses; *L1* = *L1‐ORF2*. Number of tumors and % of the HGSOC cohort are included: Color ID for purple square = INF‐low *ERV‐L1*low (*n* = 33; 32.3%); green square = INF‐low *ERV‐L1*high (*n* = 38; 37.2%); gold square = Triple‐I (*n* = 6; 5.9%); blue square = INF‐high *ERV‐L1*low (*n* = 11; 10.8%); red square = INF‐high *ERV‐L1*high (*n* = 14; 13.7%). Key = Z score for gene expression levels upper left. All *ERV‐L1* genes and the 22 genes indicative of the Triple‐I cluster are bracketed to the right of the heatmap; also, please see Table S1B. (C) HGSOC five inflammation *ERV‐L1* expression clusters identify different significant patient recurrence‐free survivals (RFS) linked with specific IRGS. Kaplan–Meier survival curves for RFS using JMP software are shown (Log rank *P* = 0.018). Bottom shows the number of stratified patients at risk from 0 to 66 months. Color key for each inflammation (INF), *ERV‐L1* (endogenous retroviruses); *L1* (*L1‐ORF2*) expression cluster is shown to the right. Colors match the heatmap in (B).

We next quantified the *ERV* families and the *L1‐5′UTR* and *L1‐ORF2* for gene expression levels in HGSOC (*n* = 102) compared with healthy fallopian tubes (*n* = 13) and ovarian (*n* = 16) tissues using our primer sequences. Results showed that *ERV* elements, the *L1‐5′UTR* and the *L1‐ORF2* were significantly overexpressed in tumors compared with ovarian tissues (*P* < 0.0001) (Fig. 1A). Surprisingly, the *L1‐ORF2* gene was significantly overexpressed in fallopian tubes compared with tumors (Fig. 1A). Importantly, comparing the HGSOC total gene expression levels of all three retroelements using our primers showed a hierarchy, where the *L1‐5′UTR* was the highest (mean = 137 287.57 molecules·ng^−1^ RNA), then *L1‐ORF2* (mean = 61 280.27 molecules·ng^−1^ RNA) and *ERV* total (mean = 2422.68 molecules·ng^−1^ RNA) as the lowest. The over 2‐fold higher expression levels of the *L1‐5′UTR* to *L1‐ORF2* could stem from the greater number of chromosomal locations of the *L1‐5′UTR* using our primers (Table S1E). To gain insight, if HGSOC gene expression levels of *ERV*, *L1‐5′UTR* and *L1‐ORF2* correlated with each other, we performed a Spearman correlation analysis. Strikingly, results showed that the Spearman correlation coefficient (95% CI) was highly significant between *ERV* total, *L1‐5′UTR* and *L1‐ORF2* and ranged between *r* = 0.8188 and 0.9853 (Fig. S2). To further test, if *ERV* total, *L1‐5′UTR* and *L1‐ORF2* expression correlated with genomic de‐repression, we analyzed the *CTCFL* gene, which is known to represent a hypomethylated DNA state in cancers, including HGSOC [40]. Results showed a significant positive correlation between total *ERVs*, *L1‐5′UTR* and *L1‐ORF2* expression with *CTCFL* (*P* < 0.0001) (Fig. S2). Taken together, we interpret our above findings that overexpression of *ERVs*, *L1‐5′UTR* and *L1‐ORF2* are highly expressed and closely linked in HGSOC, which can be partly explained due to DNA hypomethylation [6, 15, 40, 44].

### 3.2 Five distinct *ERV‐L1‐ORF2* inflammation HGSOC clusters determine survival

HGSOC classifies mainly as ‘cold’ or uninflamed tumors with reduced numbers of TILs, in contrast to ‘hot’ or inflamed tumors [45, 46]. However, higher numbers of TILs can be found in a fraction of HGSOC, resulting in increased patient survival, supporting antitumor activity [32]. Inflamed tumors enriched with T cells associate with inflammation gene signatures, where some tumors overexpress the immune checkpoint ligand PD‐L1, a marker for tumor immune evasion [45]. We first tested if high levels of *ERVs* and *L1‐ORF2* (signified as *L1*) gene expression positively correlated with high inflammation and better survival of patients with HGSOC and vice versa. Implementing our NanoString gene panel, we chose 99 genes (Inflammation and Regulatory Gene Signature = IRGS) correlating with the immune response (Fig. 1B, Table S1B). The IRGS included nucleic acid sensors, IFN pathway members, immune cells, cytokines, and other immune regulators. Some selected genes overlapped with previously published inflammation gene signatures [45]. Using an unsupervised hierarchical clustering analysis and correlating HGSOC (*n* = 102) *ERV‐L1* gene expression with the IRGS, we identified five distinct tumor clusters with significantly different patient recurrence‐free survivals (RFS; 5.5‐year) (*P*: 0.018) (Fig. 1B,C, Table 1). As predicted, one cluster showed high inflammation (inflammation‐high) with high *ERV‐L1* expression (*ERV‐L1*high) (13.7%), which positively correlated with longer patient survival (Fig. 1B,C, Table 1). Unexpectedly, a smaller tumor cluster with high inflammation but low *ERV‐L1* gene expression (5.9%) significantly demonstrated the best patient survival. This unique tumor cluster exhibited high gene expression of 22 genes, including immune checkpoint regulators, like *CTLA4*, *TIGIT*, *LAG3*, and *CD27*, immune cell surface markers, and inflammatory genes (Fig. 1B,C and Table 1, Table S1B). We termed this cluster Triple‐I, for high Immune cells, Inflammation, and Immune checkpoints.

In contrast, the remaining three tumor clusters showed the worst patient survival (Fig. 1B,C and Table 1). One tumor group signified low inflammation with low *ERV‐L1* expression (inflammation‐low *ERV‐L1*low) (32.3%), supporting possible *ERV* element repression, like methylation and lower immune cell antitumor activity. The largest tumor cluster unexpectedly showed low inflammation with high *ERV‐L1* gene expression (inflammation‐low *ERV‐L1*high) (37.2%), suggesting a block initiating inflammation despite increased *ERV‐L1* expression. The last tumor cluster was associated with high inflammation and low *ERV‐L1* expression (inflammation‐high *ERV‐L1*low) (10.8%). All of the above analyses show the intrinsic and complex nature of HGSOCs in the context of *ERV‐L1* and inflammation gene expression relating to patient survival.

Following the identification of five significant inflammation *ERV‐L1* HGSOC clusters associating with diverse survivals, we further tested these clusters with clinical and pathological variables using a Cox Regression analysis (Table 1, Table S1H,I). The following variables were analyzed and multivariably adjusted, like the application of adjuvant chemotherapy, pT‐stage, resection status (R), pN‐stage (lymphonodal stage) and lymphovascular invasion. Results showed that only the pT3 versus pT1, chemotherapy (ACT) and the tumor cluster phenotypes were significant independent factors for survival (Table S1H). Importantly, the Triple‐I group remained as an independent predictor of improved RFS when compared to inflammation‐low *ERV‐L1*low (HR = 0.09; *P* = 0.002), inflammation‐low *ERV‐L1*high (HR = 0.09; *P* = 0.002) and inflammation‐high *ERV‐L1*low (HR = 0.14; *P* = 0.038) (Table S1H). Additionally, the Triple‐I group also remained as an independent predictor of improved RFS by re‐analyzing a multivariably adjusted Cox regression for the Triple‐I group versus all other cluster groups together (HR = 0.14; *P* = 0.0127) (Table S1I).

**Fig. 2:**
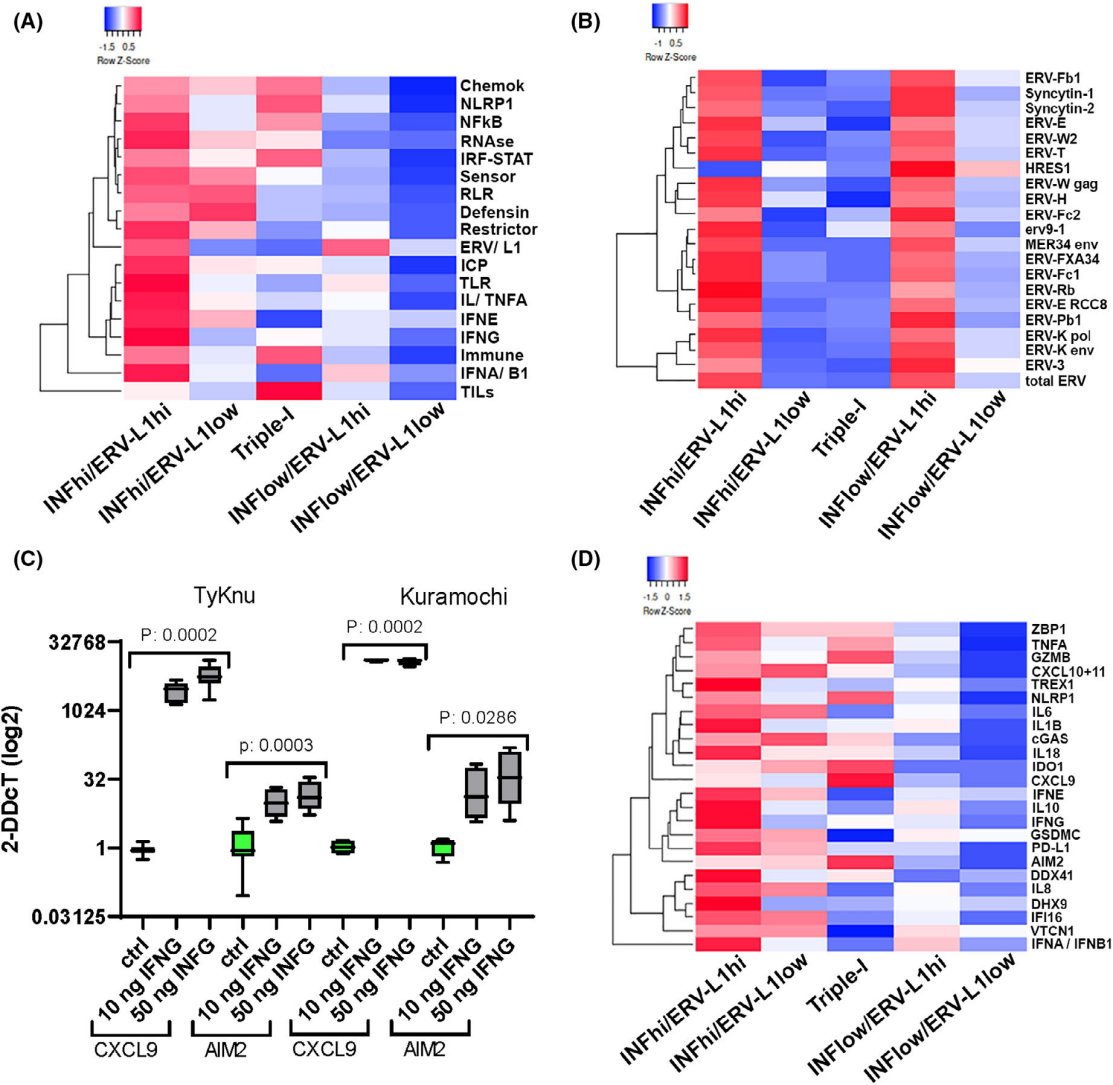
Heatmaps show differences of *ERV‐L1* expression levels and or Inflammation regulatory gene signatures (IRGS) between the five tumor clusters (please see Table 1 for tumor number and % representing the inflammation *ERV‐L1* clusters, and Table S1B for the IRGS genes). Inflammation (INF); *ERV* = endogenous retroviruses and *L1* (*L1‐ORF2*). (A) Heatmap of the IRGS with some additional genes representing various inflammatory functions with the five tumor clusters. NanoString counts in log2 of different gene expression levels. Tumor clusters: From Top to bottom: **ChemoK** = *CCL3,4; CXCL8,9,10,11; CXCR4*; ***NLRP1***; **NFKB** = *NFKB1/2, TBK1*; **RNAse** = *RNAseH1, RNAseH2A, RNAseL, XRN1,2, DICER, DROSHA*; **IRF‐STAT** = *IRF1,2,3,5,7,8,9; STAT1,3*; **Sensor** = *cGAS (MB21D1); IFI16; AIM2; DDX41; STAU1/2; OAS1; OASL; DHX9; STING1 (TMEM173); ZBP1*; **RLR** = *MAVS; RIG‐I (DDX58); DHX58; IFIH1*; **Defensin** = *IFI27,35,44,44L; ISG15,20; SETDB1; KAT5*; **Restrictor** = *APOBEC3A,3B; ADAR1; SAMHD1; TRIM22,28,40; TREX1; IFI6; MOV10; MX1,2; ZC3HAV1; RIF1; IFITM1,3*; **ICP** = immune checkpoints = *LAG3, CTLA4, PDCD1LG2 (PD‐L2), PDCD1 (PD‐1), CD274 (PD‐L1), CD27, HAVCR2 (TIM‐3); TIGIT, VTCN1, NECTIN4*; **IL** + **TNFA** = *IL6,10,18,1B*, *TNFA*; **TLR** = *TLR3,4,9*; ***IFNE*; *IFNG;* Immune** = immune cells CD3z (CD247); *CD4; CD8A; CD44; CD68; CD80; CD163; FOXP3; GZMB; PRF1; NKG7; EOMES*; ***IFNA,B1*** and **TILs** = sTILs in % analyzed by H&E for each tumor cluster: INF‐high *ERV‐L1*high: 11.92%; INF‐high *ERV‐L1*low: 8.9%; Triple‐I: 22.83%; INF‐low *ERV‐L1*high: 9.5%; INF‐low *ERV‐L1*low: 3.3%. (B) Heatmap of absolute quantification real‐time PCR results for single *ERVs* and the total *ERV* element expression levels (bottom right) for each inflammation *ERV‐L1* cluster stated below. (C) *CXCL9* and *AIM2* gene expression induction after IFNG treatment of high‐grade serous ovarian carcinoma (HGSOC) cell lines. *Y*‐axis = 2‐DDcT gene expression values (log2) of *CXCL9* and *AIM2*; *X*‐axis = IFNG treatment (21 h) of TyKnu and Kuramochi HGSOC cell lines; untreated control (ctrl) (green), 10 ng·mL^−1^, 50 ng·mL^−1^ IFNG (gray), *n* = 6. Shown are box and whiskers with minimum to maximum values. Significance was calculated using the two‐tailed Mann–Whitney *U* tests. (D) Heatmap of NanoString counts in log2 of 26 different gene expression levels relating to single genes from the IRGS with a highlight of pyroptosis related genes.

**Fig. 3:**
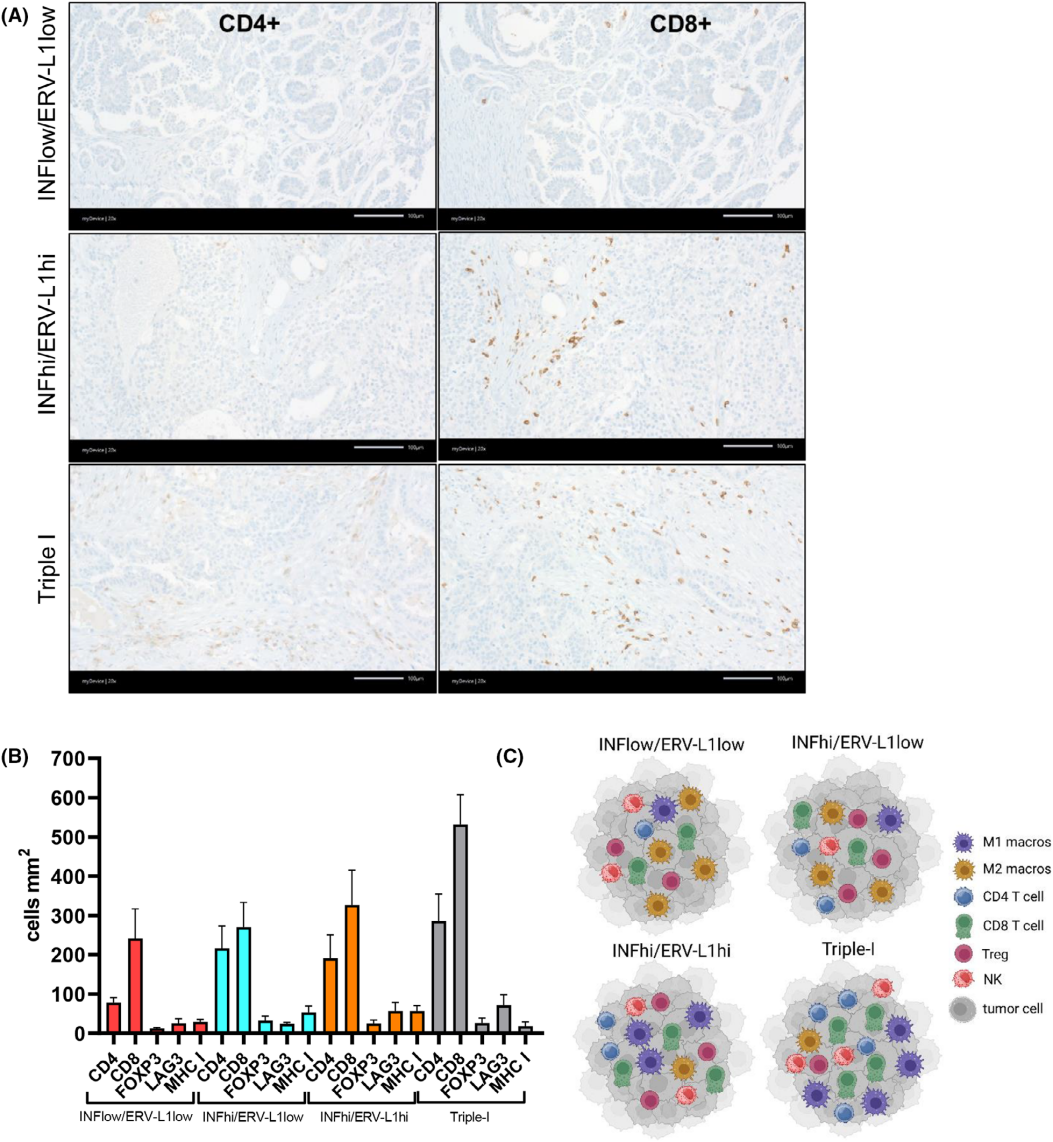
High‐grade serous ovarian carcinoma (HGSOC) inflammation *ERV‐L1* gene expression clusters and association with immune cells; Inflammation = INF, *ERV* = endogenous retroviruses, *L1 = L1‐ORF2*. (A) HGSOC tissues from inflammation *ERV‐L1* gene clusters (see Table 1 for tumor clusters) were hybridized with CD4 and CD8 antibodies using IHC. These represent biological‐independent replicates from inflammation‐low *ERV‐L1*low tumors (*n* = 33); inflammation‐high *ERV‐L1*low (*n* = 11); inflammation‐high *ERV‐L1*high (*n* = 14) and Triple‐I tumor clusters (*n* = 6 tumors). Microscopic photographs of IHC show CD4^+^ and CD8^+^ immune cells from three tumor clusters representing inflammation‐low *ERV‐L1*low (top); inflammation‐high *ERV‐L1*high (middle) and Triple‐I tumor clusters (bottom). Bars in the lower right indicate 100 μm. (B) Graph showing quantification of immune cells using qpath software (cells·mm^−2^) for specific tumor clusters (see Table 1 for number of tumors and % of each tumor cluster; *n* = X of tumors quantified per tumor cluster) inflammation‐low *ERV‐L1*low (*n* = 33 tumors in this cluster); inflammation‐high *ERV‐L1*low (*n* = 11 tumors in this cluster); inflammation‐high *ERV‐L1*high (*n* = 14 tumors in this cluster) and Triple‐I tumor clusters (*n* = 6 tumors in this cluster), which also exemplifies (A) and (C). Graph bars represent SEM. (C) Schematic diagrams show the hierarchy of immune cells of four representative inflammation *ERV‐L1* tumor clusters. Immune cell amounts (%) calculated using cibersort software, which implements many gene expression variables, and was confirmed in part using q‐path software for quantification of IHC expression of CD4^+^, CD8^+^ and Treg (FOXP3). CIBERSORT quantification of M1 (CD68) and M2 (CD163) macrophages, NK cells and Tregs among Fallopian tubes (*n* = 13) = M1, M2 = 0%; NK = 1%; Treg = 4% (not shown here); and four tumor clusters: inflammation‐low*/ERV/L1*‐low (*n* = 33): M1 = 5%; M2 = 12%; NK = 7%, Treg = 12%; inflammation‐high*/ERV/L1*‐low (*n* = 11): M1 = 25%; M2 = 10%; NK = 5%, Treg = 18%; inflammation‐high*/ERV/L1‐*high (*n* = 14): M1 = 8%; M2 = 2%; NK = 8%, Treg = 17%; Triple‐I (*n* = 6): M1 = 8%; M2 = 4%; NK = 11%, Treg = 7% (see Table 1 for number of tumors and % of each tumor cluster; *n* = X of tumors quantified per tumor cluster).

In order to gain additional insight into the molecular nature of the five unique tumor clusters with patient survival, we analyzed in more detail each cluster in terms of *ERV‐L1* expression with the IRGS (Table S1B). This analysis also included immune cells quantified using (a) CIBERSORT [43], which computes different immune cells from gene expression profiles; (b) qupath software for quantifying IHC protein expression hybridization signals; and (c) H&E for determining the percentage of sTILs. Regarding the inflammation‐high *ERV‐L1‐*high tumor cluster, results showed that high expression of *ERV‐L1*, except *HRES1*, was associated with an innate immune response (Fig. 2A,B). For example, the induction of classical IFN type I signaling via dsRNA possibly stemmed from *ERV* sense and antisense transcripts [6], *L1* bidirectional transcripts, or RNA:DNA hybrids [21, 23]. Results showed high expression of (a) cytosolic and membrane *TLR3, 4, 9*; (b) sensors for dsRNA, for example, *MDA5*, *RIG‐I*; or for *dsDNA*, for example, *cGAS* or the Z‐form of RNA or DNA, for example, *ZBP1*; (3) ISGs, like *IFI16*, *AIM2*, *OASL*; interferon regulatory factors (*IRF1‐9*); (d) transcription factors *NFKB1* and *NFKB2*; (e) pathway restrictor regulators *ADAR1*, *SAMHD1*; (f) immune cells, immune checkpoint regulators, and chemokines (*CCL3*, *CCL4*, and *CXCL8‐11*) and (g) high sTILs (Fig. 2A). Along with moderate amounts of CD4^+^ and CD8^+^ immune cells, importantly, M1 macrophages were also enriched in the inflammation‐high *ERV‐L1‐*high cluster, whereas M2 macrophages were lower (Fig. 3A–C). All of the above is in line with better patient survival (Fig. 1C).

The Triple‐I cluster with the best patient survival (Fig. 1B,C) supports a role for IFNG mediated inflammation. High IFNG leads to higher *STAT* and target gene expression, for example, *AIM2*, *CXCL9*, and *IRFs* and in addition, low expression of *IFNA* or *B1* (Fig. 2A). High inflammation in context with low *ERV* expression is novel due to an absence of viral mimicry (Fig. 2B). Additionally the highest amount of sTILs (22.83%) in the Triple‐I cluster was corroborated by the highest expression of immune cell surface markers (Figs 2A and 3A–C). Importantly, we proved with two HGSOC cell lines TyKnu and Kuramochi that upon addition of IFNG both *AIM2* and *CXCL9* significantly increased (Fig. 2C). These findings demonstrate that HGSOC cells represent one source for high *AIM2* and *CXCL9* gene expression levels found in the Triple‐I cluster, where both genes represent IFNG targets. For Triple‐I tumors, in addition to IFNG mediated cytotoxic T‐cell tumor killing involving genes, like GZMB, high *AIM2* along with *NLRP1* expression support a role of pyroptosis via inflammasomes (Fig. 2D).

**Fig. 4:**
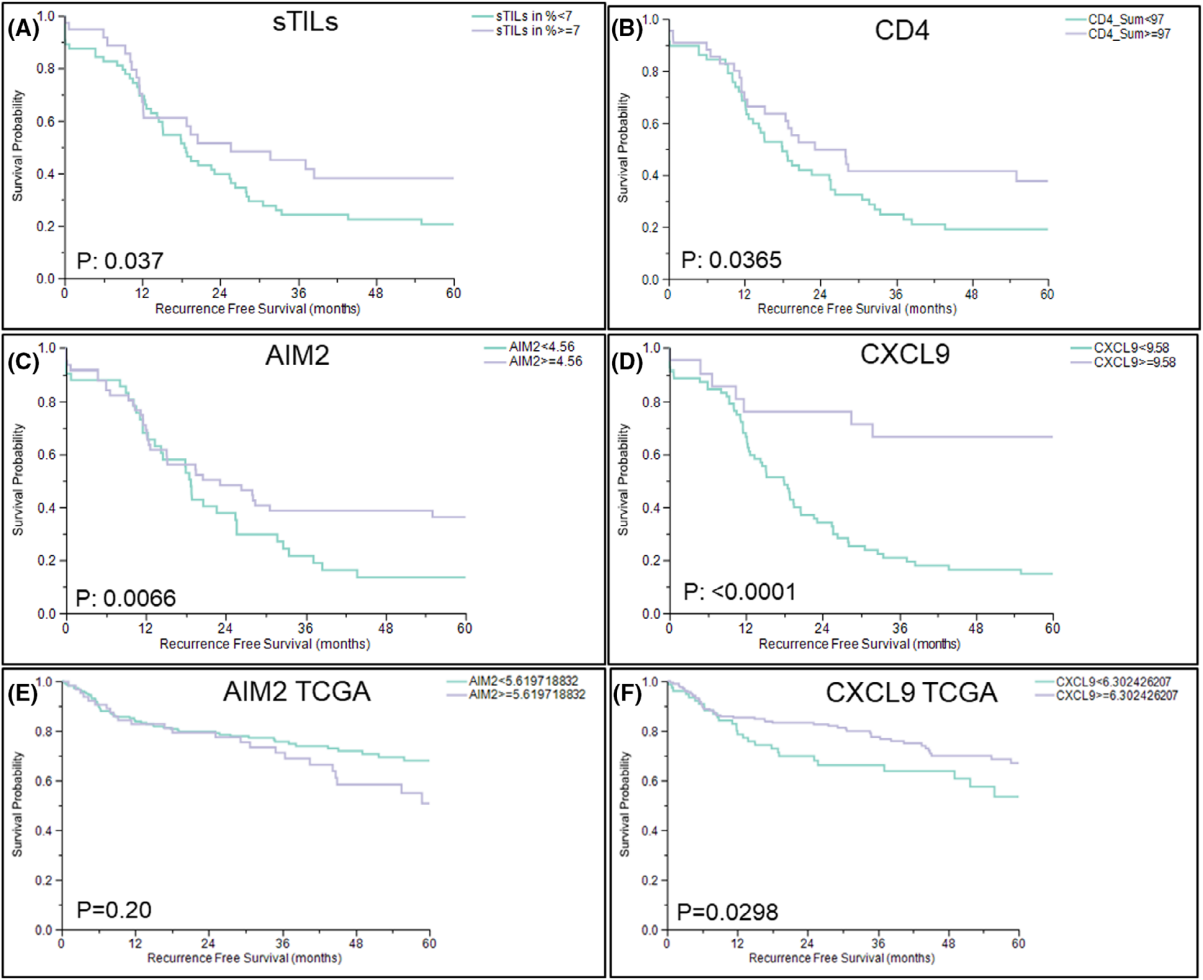
Landmark TILs and genes for significant recurrence‐free patient (RFS) survival of high‐grade serous ovarian carcinoma (HGSOC) (*n* = 102) and TCGA analyses with HGSOC (*n* = 294). Kaplan–Meier survival curves for RFS using jmp computer software with our HGSOC cohort. (A–D) and TCGA (E, F); (A) sTILs; (B) CD4^+^; (C) *AIM2*; (D) *CXCL9*; (E) TCGA (*n* = 294) *AIM2*; (F) TCGA (*n* = 294) *CXCL9*.

We then assessed our HGSOC cohort using other specific variables associating with patient survival in order to corroborate our above findings of both the inflammation‐high *ERV‐L1*high and Triple‐I tumor clusters. Significant longer patient survivals associated with higher amounts of sTILs (*P*: 0.037) and CD4^+^ T cells (*P*: 0.0365) (Fig. 4A,B), which are supported by the literature [47]. Better survival significantly correlated with higher gene expression of *AIM2* (*P*: 0.0066, Fig. 4C) and *CXCL9* (*P* < 0.0001, Figs 2D and 4D). Using a ‘pruning’ statistical test, high *CXCL9* expression identified the Triple‐I cluster with a precision of 100%. In order to test the significance of the Triple‐I cluster, we analyzed an HGSOC cohort from TCGA (*n* = 294) using a panel of the 22 highest overexpressed genes specific for this cluster (Fig. 1B,C; Table S1B). Remarkably, results demonstrated that *CXCL9* was the main contributor for survival, especially of the HGSOC Triple‐I cluster (*P*: 0.0298, Fig. 4F), whereas *AIM2* showed no significance for the TCGA cohort.

In contrast to the above, regarding the ‘cold’ inflammation‐low *ERV‐L1* low tumor cluster, all *ERV‐L1* genes and the IRGS showed no induction, except the single *ERV HRES1* induced to higher levels (Fig. 2A,B,D). On the contrary, the largest ‘cold’ inflammation‐low tumor cluster was associated with high *ERVs* (inflammation‐low *ERV‐L1*high; 37.2%). Although *ERV* expression levels were high, being comparable to the inflammation‐high *ERV‐L1*high cluster, we examined for possible aberrant dsRNA sensor expression (Fig. 2A,B; Fig. S3). Indeed, gene expression of both *MDA5* and *RIG‐I* dsRNA sensors was significantly lower in the inflammation‐low *ERV‐L1*high tumor cluster, supporting an aberrant block in IFN signaling (Fig. S3). Importantly, low dsRNA sensor expression was comparable to the inflammation‐low *ERV‐L1*low and Triple‐I tumors, indicating no signaling due to *ERV* and *L1* dsRNA. However, this was in contrast to the inflammation‐high *ERV‐L1*high cluster, where MDA5 and RIG‐I receptors were high and showed an IFN response.

In light of low *ERV‐L1* gene expression in the smaller ‘hot’ Inflammation‐high *ERV‐L1*low tumor cluster (10% of all HGSOC) with poor patient RSF survival, we noted high *MDA5* and *RIG‐I* levels (Fig. S3). Although we did not examine short interspersed elements (SINE) like *Alu* RNA, which bind to RIG‐I inducing inflammation, our data could support a possible role for *Alu* RNAs or other repetitive elements mediating inflammation regarding this cluster. Furthermore, we determined differences between the inflammation‐high *ERV‐L1*low and the Triple‐I clusters, which could help to explain their opposite patient survivals (Fig. 1C). Results revealed that: (a) the Triple‐I group had higher sTILs (22.8% vs. 8.9%) and NK cells (11% vs. 5%) when compared to the inflammation‐high *ERV‐L1*low cluster, respectively; (b) the inflammation‐high *ERV‐L1*low cluster showed a higher inhibitory Treg cell content than the Triple‐I group (18% vs. 7%), respectively (Fig. 3B,C; CIBERSORT); and (c) the Triple‐I group showed a 15.7‐fold lower expression level of the immune checkpoint gene *VTCN1* (also called *B7‐H4*) when compared to the inflammation‐high *ERV‐L1*low tumor cluster (Fig. 2D).

Lastly, to determine a minimal decisive global *ERV‐L1* signature in order to discern between *ERV‐L1*high and *ERV‐L1*low with high or low inflammation, respectively, we selected *ERVs* with the highest differential expression (Fig. 2B). Results showed that an expression signature of *ERV3*, *ERV‐Kenv/pol*, *ERV‐H*, and *ERV‐Wgag*, as well as *L1* was sufficient.

### 3.3 Immune checkpoint receptors and novel ligand interactions within tumor clusters

Programed death receptor 1 (PD‐1) is expressed on T cells following antigen presentation and T‐cell activation in order to attenuate and control the response. Inhibitory regulation occurs following PD‐1 binding to the ligands PD‐L1 or PD‐L2, expressed on antigen presenting cells (APCs) or with tumor cells leading to evasion [48]. In order to determine a role of PD‐1 and PD‐L1 protein in the HGSOC TIME, we quantified their protein expression. For all five HGSOC clusters, PD‐1 and PD‐L1 protein expression exclusively stemmed from immune cells, with no detectable PD‐L1 protein expression on tumor cells (Fig. S4A–C). This result supports no tumor PD‐L1 adaptation leading to T‐cell exhaustion in the TIME. Both the Triple‐I and the Inflammation‐high *ERV‐L1* high tumor clusters showed the highest levels of immune cells with PD‐1 and PD‐L1 protein expression at the invasion front, whereas all other tumor clusters were lowly expressed for both checkpoints. We reasoned that higher PD‐1 membrane expression on T cells along with increased PD‐L1 APC levels reflects normal immune cell homeostasis, resulting in a T‐cell population with an increased antitumor response, thus contributing to longer patient survival (Fig. 1C). High PD‐L1 expression on immune cells associating with better patient survival is supported by the literature [41, 47].

**Fig. 5:**
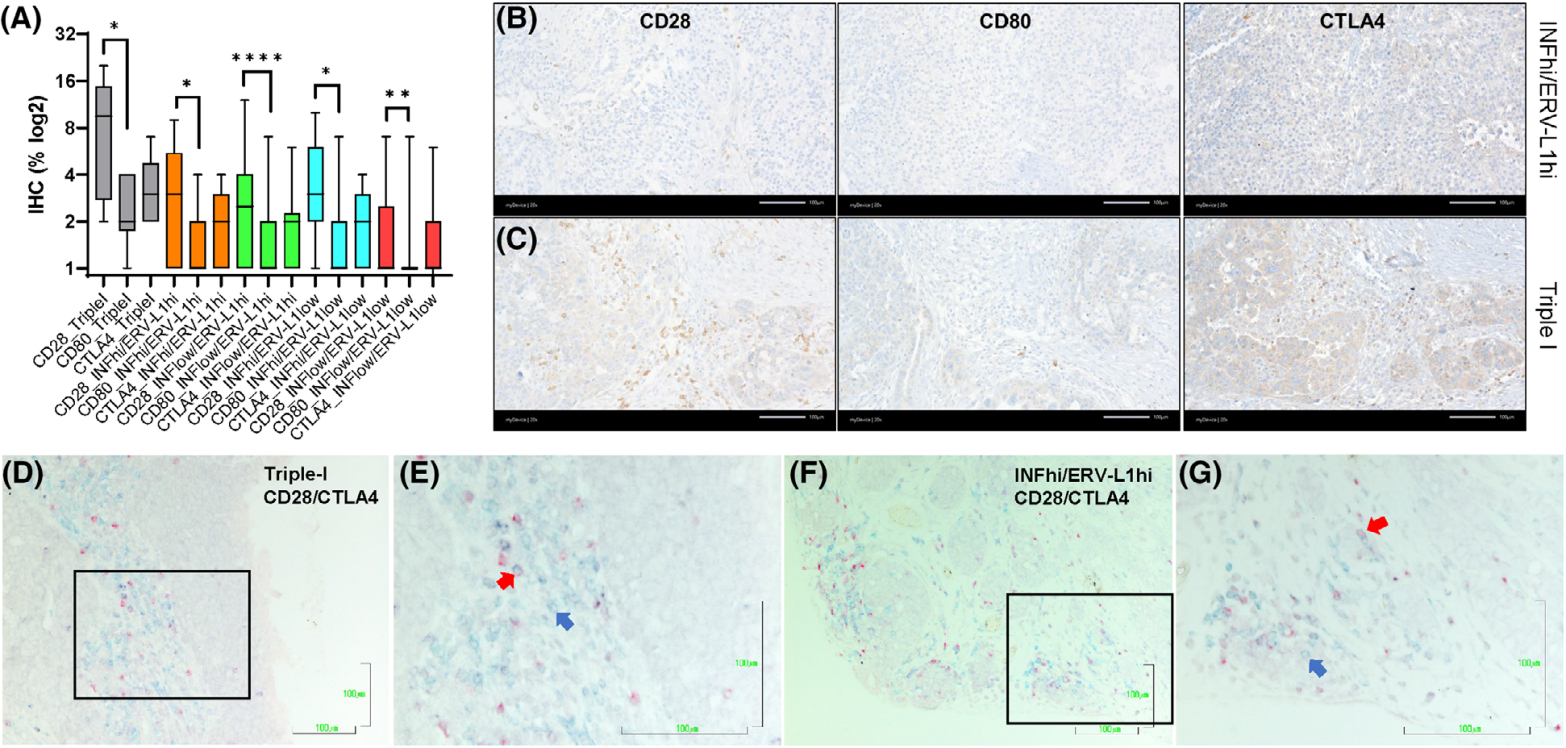
Comparison of protein expression of immune checkpoint regulators (CD28, CD80, and CTLA4) between the five inflammation *ERV‐L1* clusters in high‐grade serous ovarian carcinoma (HGSOC) (*n* = 102 tissues) using IHC. (A) Graph represents IHC quantified using q‐path software of CD28, CD80, and CTLA4 (% cells log2) with a comparison of all five inflammation *ERV‐L1* tumor clusters with significant differences according to a two‐tailed Mann–Whitney *U*‐test: Triple‐I (*n* = 6 biologically independent replicates) CD28 vs. CD80: *P: 0.0476; inflammation‐high *ERV‐L1*high (*n* = 14 biologically independent replicates) CD28 vs. CD80: *P: 0.0103; inflammation‐low *ERV‐L1*high (*n* = 38 biologically independent replicates) CD28 vs. CD80: ****P < 0.0001; inflammation‐high *ERV‐L1*low (*n* = 11 biologically independent replicates) CD28 vs. CD80: *P: 0.0281; inflammation‐low *ERV‐L1*low (*n* = 33 biologically independent replicates) CD28 vs. CD80: **P: 0.0049. Shown are box and whiskers with minimum to maximum values. (B, C) Representative photographs from two tumor clusters (inflammation‐high *ERV‐L1*high and Triple‐I) show IHC expression of CD28, CD80 and CTLA4. (D–G) Multiplex IHC using two color detection of CD28 and CTLA4 was performed with primary tumor tissues from the Triple‐I cluster (*n* = 6 biologically independent replicates) and the inflammation‐high *ERV‐L1*high cluster (*n* = 3 biologically independent replicates). Photographs show examples of CD28 (green/blue color) and CTLA4 (red) of the primary tissues from the Triple‐I and inflammation‐high *ERV‐L1*high tumors. Squares in (D) and (F) represent magnifications of (E) and (G). All scale bars are indicated in the lower right as 100 μm. See Fig. 5G for quantification of CD28 and CTLA4 protein expression.

The CD28 receptor is constitutively expressed on T cells and, following antigen presentation, results in a fully active state leading to clonal expansion, cytokine, and enzyme secretion necessary to target virally infected or tumor cells [49]. To negatively regulate T‐cell activation, the cytotoxic T lymphocyte associated protein 4 (CTLA4 receptor) becomes co‐expressed with CD28 at the membrane. Due to similar amino acid homologies, both receptors compete for the same CD80 ligand on APCs [49]. This T‐cell attenuation occurs at early stages of T‐cell activation following antigen presentation, for example, in the lymph nodes. High gene expression of the CTLA4 receptor is a prominent characteristic of the Triple‐I tumor cluster (Figs 1C and 2A, Fig. S5). In order to discern CTLA4 cytosolic or membrane localization, along with its ratio to CD28, we quantified and compared protein levels of CTLA4, CD28, and the ligand CD80 in the lymph nodes and in the TIME between the same patients representing specific tumor clusters (Fig. S5A–E). Comparing lymph node tissues demonstrated that CD28 was the highest significantly expressed checkpoint in the lymph node compared with CD80 and membrane CTLA4, which had equal amounts using IHC (Fig. S5A,D,E). These results support T‐cell activation following antigen presentation occurring in lymph nodes for all three tumor clusters. However, comparing patient‐matched tumor tissues showed CD28 was the highest expressed in the TIME for the Triple‐I cluster compared with the other tumor clusters (Fig. S5B). The correlation of protein expression was confirmed within the TIME among all five HGSOC tumor clusters (*n* = 102) (Fig. 5A–C). Since CD28 represents an activated T‐cell state, we performed multiplex IHC to address the ratio of CD28: CTLA4 co‐expression on immune cells (Fig. 5D–G, Fig. S5C). Results showed both clusters with the highest inflammation (Triple‐I and Inflammation‐high *ERV‐L1* high) exhibited a large immune cell population in the TIME, which solely expressed CD28 without CTLA4 protein expression. These results support T‐cell activation, where CD28 binds to CD80 with no inhibitory competition mediated by CTLA4. In line with the best patient survivals (Fig. 1C), we propose that in response to tumor antigens within the lymph nodes, the activating checkpoint regulator CD28, as well as possibly others, represent a more activated T‐cell state in the HGSOC TIME, especially regarding the Triple‐I cluster (Fig. 5F).

## 4 Discussion

Since the discovery of viral mimicry via *ERVs* igniting the type I IFN response in a variety of tumors, including HGSOC, a wealth of knowledge regarding the importance of retroelements and innate immunity has been demonstrated [6, 50]. Reactivation of *ERV* element expression resulting in higher dsRNA levels within tumor cells is a canonical pathway igniting inflammation and predicts better patient survival [6, 7, 29, 51, 52]. Our present study helps to define *ERV* and *L1* gene expression levels and their contribution to inflammation in the HGSOC TIME important for patient survival using tumor tissues prior to treatment. Examination of 102 HGSOC in context with *ERV‐L1* expression and inflammation demonstrated the majority of tumors were ‘cold’ or noninflamed (69.5%), but interestingly associated with low *ERV‐L1* or high *ERV‐L1* expression linked with poorer survival. These ‘cold’ tumors revealed a significantly lower IRGS expression, especially highlighting the importance of dsRNA sensors *MDA5* and *RIG‐I*, lower sTILs, for example, CD4^+^, and a stronger association with M2 macrophages. Even despite high *ERV‐L1* expression noted in the ‘cold’ inflammation‐low *ERV‐L1*high tumor cluster, we found aberrant low levels of *MDA5* and *RIG‐I* supporting an adaptive tumor response within the TIME due to a block igniting inflammation. Recent findings from the literature also point to other deregulated genes involved in IFN signaling, which contribute to tumor adaptive responses. For example, the *DHX9* helicase identified as a repressor for both dsRNA and dsDNA induced by DNA damage was aberrantly overexpressed in small cell lung cancer [53]. However, we found no significant expression differences of *DHX9* between all five tumor clusters in our HGSOC cohort.

The discovery of two distinct ‘hot’ inflamed tumor clusters (total 19.6%) within our HGSOC cohort with the best patient survivals, but with opposing high or low *ERV‐L1* expression is novel and supports different IFN signaling. For example, it is striking that the inflammation‐high *ERV‐L1*high cluster representing 13.7% of HGSOC demonstrated the most enhanced dsRNA sensors, *IFNA*, *IFNB1*, and *IFNG* signaling. Together this pathway resulted in high levels of ISGs, cytokines, and sTILs and supports an innate immune response. On the other hand, we propose that IFNG plays a more regulatory role in the Triple‐I cluster with the highest number of sTILs and CD4^+^ T cells and the lowest *ERV‐L1* expression, supporting an adaptive immune response. These results speak for a direct role of T‐cell killing of tumor cells, which we support is due to increased tumor antigens and T‐cell expansion stemming from lymph nodes. Further support for highly active cytotoxic T cells stems from our finding that CD28 was solely expressed on a high percentage of immune cells without the negative regulator CTLA4 in the TIME. Cytotoxic CD4^+^ T cells have been identified in cancers and associated with GZMB and Perforin (PRF) expression via the transcription factor EOMES [54]. Importantly, we found *EOMES* highly upregulated in the inflammation‐high *ERV‐L1‐*high cluster and *GZMB* and *PRF* with the Triple‐I tumor cluster (Figs 1B and 2A).

In line with the above, specifically *CXCL9* is highly expressed in both inflammation‐high *ERV‐L1‐*high and Triple‐I clusters. However, it is evident that the highest *CXCL9* expression significantly represents a main contributor to the best survival in the Triple‐I group as well as with the TCGA analysis. One explanation could be that this cytokine attracts activated and memory T cells, suppressing tumor growth [55]. Previously, it was shown that high CXCL9 and CXCL10 protein expression increased the survival of HGSOC patients and positively associated with TILs [56]. This study also showed that CXCL9 protein secretion occurred from ovarian cancer cell lines after the addition of IFNG and TNFA using cell culture, which is substantiated by our findings that *CXCL9* as well as *AIM2* are target genes for IFNG. TILs and M1 macrophages secrete CXCL9 [57], but the above findings support that tumor cells, especially from the Triple‐I cluster, also secrete *CXCL9* in the TIME, leading to further TIL recruitment. This scenario could explain our findings of the highest sTILs and M1 macrophages associated with the Triple‐I tumor cluster.

Ignition of the IFN pathway is also critical for initiating DNA damage and apoptosis resulting in tumor cell death in the TIME. In addition to apoptosis, pyroptosis describes inflammatory cell death with formation of inflammasomes, cell membrane breaks with dsDNA, and IL18 and IL1B release [4]. The main genes involved in inflammasomes are *AIM2*, *GSDMs*, *NLR*, and *Caspases*. This signaling network hints at a beneficial regulation of sensors, inflammation, and apoptosis or pyroptosis. A study analyzing pyroptosis genes from a TCGA cohort of ovarian cancer patients demonstrated that *AIM2* was upregulated in ovarian tumors and contributed to a longer overall survival [58]. We found support for inflammasomes in Triple‐I tumors due to our findings that *AIM2* and *NLRP1* were the highest expressed genes compared with all clusters (Fig. 2D). In addition to AIM2 and NLRP1, cGAS and IFI16 also bind dsDNA [59, 60]. The latter dsDNA sensors are ISGs, except NLRP1, where *IFI16* and *AIM2* become induced via IFNG [61], as corroborated with our HGSOC cell culture studies. In contrast to the inflammation‐high *ERV‐L1*high cluster, the Triple‐I cluster showed lower *IFI16* expression, which could augment the inflammasome, due to the IFI16B protein, which inhibits AIM2 binding to dsDNA and attenuates an active inflammasome [62]. In regard to pyroptosis and contributory IFN signaling members, it should be noted that the expression profile of the inflammation‐high *ERV‐L1*high and the Triple‐I clusters are partly in contrast. For example, *IDO1*, *AIM2*, *CXCL9*, *NLRP1*, and *GZMB* showed the highest expression in the Triple‐I cluster, whereas *IL8*, *IL6*, *GSDMC*, *IFNA*, and *IFNB1* are prominent in the inflammation‐high *ERV‐L1‐*high cluster (Fig. 2D).

Lastly, our results demonstrate that the hierarchy of overexpression and significant correlations of *L1‐5′UTR*, *L1‐ORF2*, and *ERVs* in HGSOC due to DNA hypomethylation point to their significance in this cancer entity. It is important that a recent study found that 53% of cancer patients had somatic retrotranspositions due to reactivated L1 [63]. For ovarian cancer, *L1* reactivation led to mutations and genome heterogeneity in ovarian cancer cell lines [64]. Although further studies are needed to unravel the complexities of *L1* retroelement contributions in cancer inflammation, esp. for L1‐ORF2, we support the idea that they represent a source for dsRNA and RNA: DNA linked with an IFN response. In addition, ERV pols (esp. ERV‐Kpols) should also be considered as an origin of RNA: DNA hybrids [65], which have to be analyzed further for their role in IFN signaling.

## 5 Conclusions

**Fig. 6:**
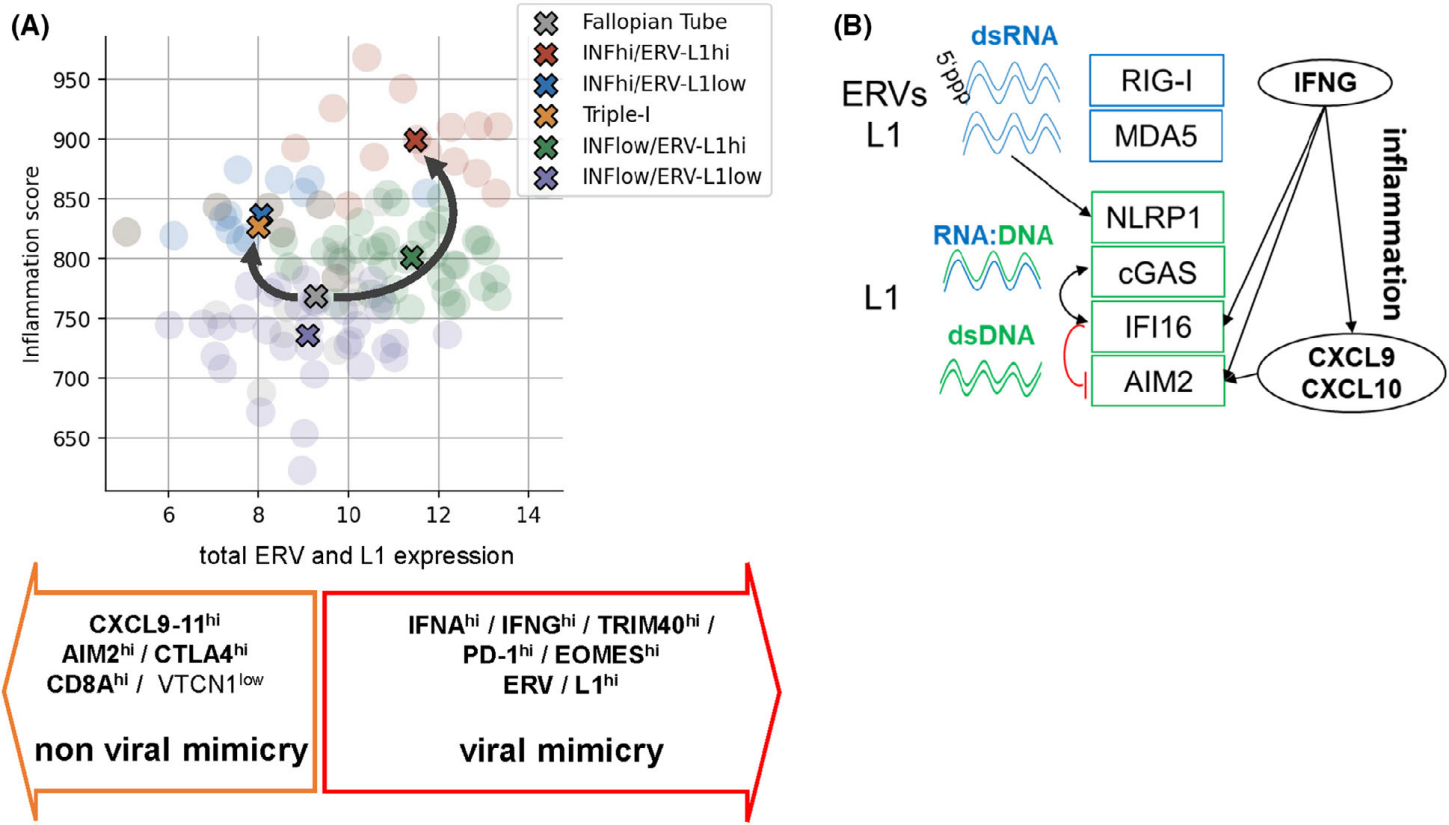
(A) Model showing viral and ‘nonviral’ mimicry regulating inflammation. *ERV‐L1* total molecules (*X*‐axis) and the inflammation score (*Y*‐axis) for each of the five high‐grade serous ovarian carcinoma (HGSOC) clusters and fallopian tubes. Please see Section 2 for all statistical explanations and Table 1 for the number and % of specific tumor clusters and fallopian tubes (*n* = 13) plotted in the graph. The inflammation score was calculated for each tumor sample from the inflammation‐low and inflammation‐high clusters as the sum of the log2‐expression levels of 99 IRGS genes. Colored circles represent single tumors within its inflammation *ERV‐L1* tumor cluster (matched colored ‘X’ in the inlay box). The inflammation score summarizes the state of HGSOC inflammation in a single continuous metric. This score was directly compared with *ERV‐L1* total expression values for each inflammation *ERV* cluster. Below in arrow‐shaped boxes are the *most significant DEGs* for nonviral and viral mimicry based on an analysis with a minimal 4‐fold expression change and an adjusted *P* < 0.01. In contrast, *VTCN1*, especially regarding the Triple‐I tumor cluster, was down regulated in nonviral mimicry. Feature pruning was used to obtain the smallest set of genes associated with the cluster representing the highest precision. In order to identify Triple‐I tumors with 100% precision only *CXCL9* was necessary. (B) Model shows a molecular regulation for different nucleic acid species (dsRNA, RNA:DNA, dsDNA) derived from ERV and L1 and recognized by their sensors leading to inflammation. In addition, we and others showed that the inflammation cytokine IFNG induces *AIM2* and its negative regulator *IFI16*, as well as other inflammation markers like CXCL9 and CXCL10.

We propose a model for HGSOC, whereby both high *ERV‐L1* and low *ERV‐L1* expression can be exemplified as two distinct inflammation pathways, with and without viral mimicry (Fig. 6A). Correlating *ERV* and *L1* total expression with the most significant differentially expressed IRGS genes allows for a direct comparison and generates inflammation scores. The above analysis demonstrates that even when *ERV‐L1* elements are highly expressed, the inflammation score can be high or low and vice versa. Our study demonstrates the molecular complexity of the HGSOC TIME, where *ERV‐L1* gene expression levels associate with IFN signaling, inflammation, checkpoint protein regulation, and antitumor activity via TILs important for patient survival. It is important to note that our HGSOC cohort of 102 patients was moderate in size, consisting of all Europeans with nationalities from Germany (*n* = 96), but also from Poland, Turkey, and Serbia (*n* = 6). Therefore, it will be essential to expand the patient number, including diverse nationalities, in order to verify each tumor cluster, especially the Triple‐I with the best patient survival. Furthermore, performing long range RNA‐seq (over 300 nt) with multiple reads for a more specific identification of retroelements as well as inflammatory genes would help to understand regulation pathways. Determining DNA and histone methylation would also help further to deduce retroelement epigenetic regulations. Taken together, we show that HGSOC consists of unique clusters, particularly one inflammatory group lacking viral mimicry associated with longer survival. We propose that these unique tumor clusters could be represented among other tumor types.

## Conflict of interest

The authors declare no conflict of interest. CM is the founder of Artifact Research GmbH.

## Author contributions

RS, ME, and PLS designed and supervised the project. LG, RS, ME, and PLS performed the experiments. ME, MWB, and AH obtained patient tissue and clinical data. ME, RS, and CM performed the final data analysis. RS and PLS wrote the original draft. All authors reviewed the article and approved the final manuscript.

## Supporting information


**Fig. S1.** (A) Genomic model of *ERV* and *L1*; (B) Identification of the best reference genes for qPCR using our HGSOC cohort; (C) Comparison of ZBP1 gene expression between NanoString and qPCR from HGSOC.

**Fig. S2.** Retrotransposon and *CTCFL* expression correlation in HGSOC.

**Fig. S3.** DsRNA sensors can be deregulated blocking inflammation.

**Fig. S4.** Immune Checkpoint regulators PD1, PD‐L1, PD‐L2 specifically associate with immune cells of HGSOC.

**Fig. S5.** Comparison of protein expression of immune checkpoint regulators (CD28, CTLA4, CD80) between lymph nodes and the HGSOC TIME from the same patients using IHC.

**Table S1.** (A) Gene panel for NanoString analyses; (B) Interferon regulatory gene signature (IRGS); (C) Primer gene sequences; (D) ERV subfamilies; (E) *ERV‐L1* (*L1‐ORF2*, *L1‐5′UTR*) analyses of the human genome; (F) The exact nucleotide localizations on each chromosome of the *L1‐5′UTR* primer pair; (G) The exact nucleotide localizations on each chromosome of the *L1‐ORF2* primer pair; (H) Multivariable COX analyses of all phenotypes, including clinical pathological variables; (I) Multivariable COX Triple‐I analyses compared with other tumor clusters.

## Data Availability

Additional data are presented as Supplementary Figures and Tables.
