# Supplementary figures and images for: Tumor clusters with divergent inflammation and human retroelement expression determine the clinical outcome of patients with serous ovarian cancer
Source: Mol Oncol. 2025 Jun 10;19(12):3750–68. doi: 10.1002/1878-0261.70067 (PMC12688164; doi:10.1002/1878-0261.70067)

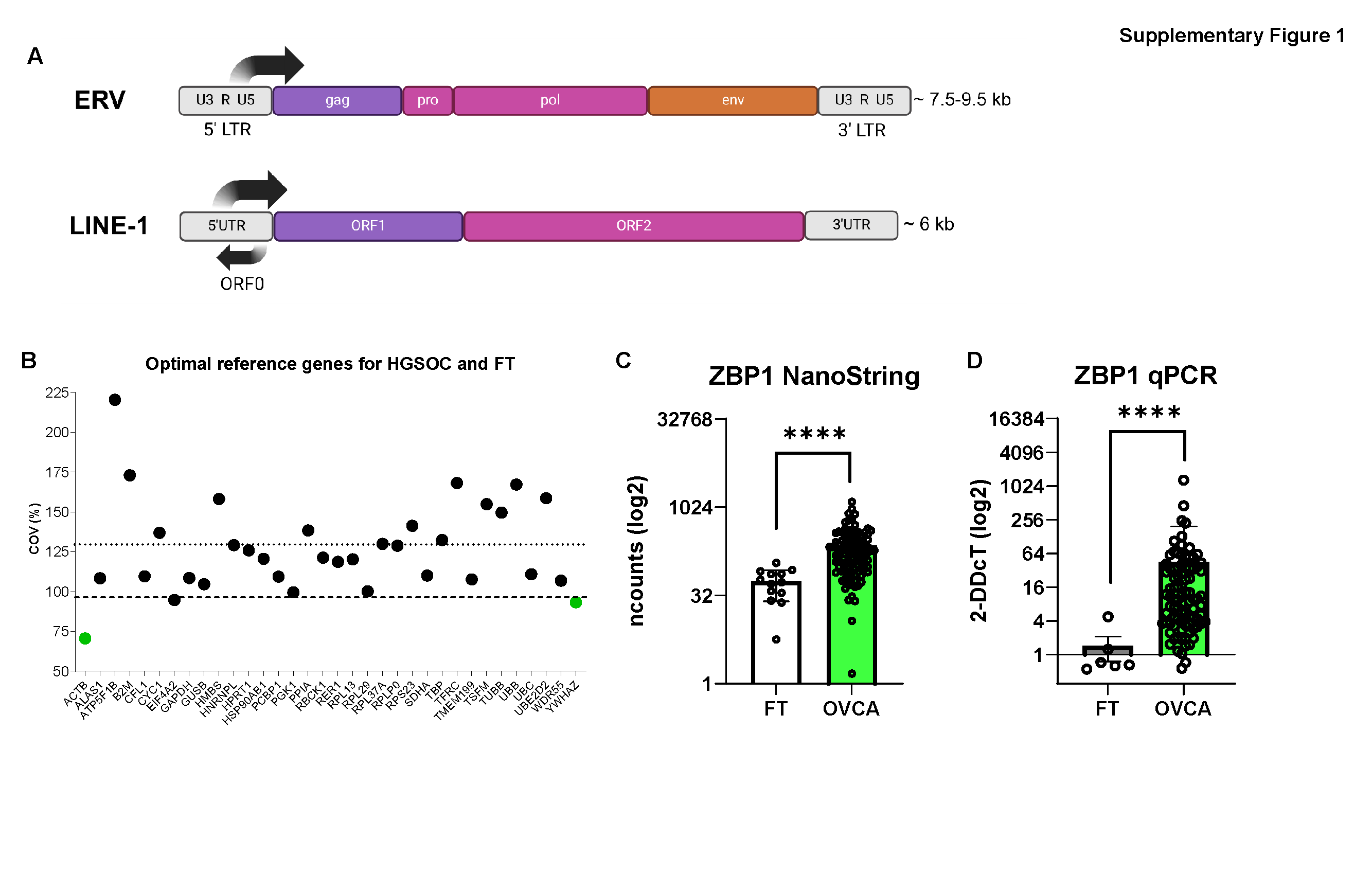

Supplement: Supplementary file 1 — Fig. S1. (A) Genomic model of ERV and L1; (B) Identification of the best reference genes for qPCR using our HGSOC cohort; (C) Comparison of ZBP1 gene expression between NanoString and qPCR from HGSOC. [file MOL2-19-3750-s002.tiff]

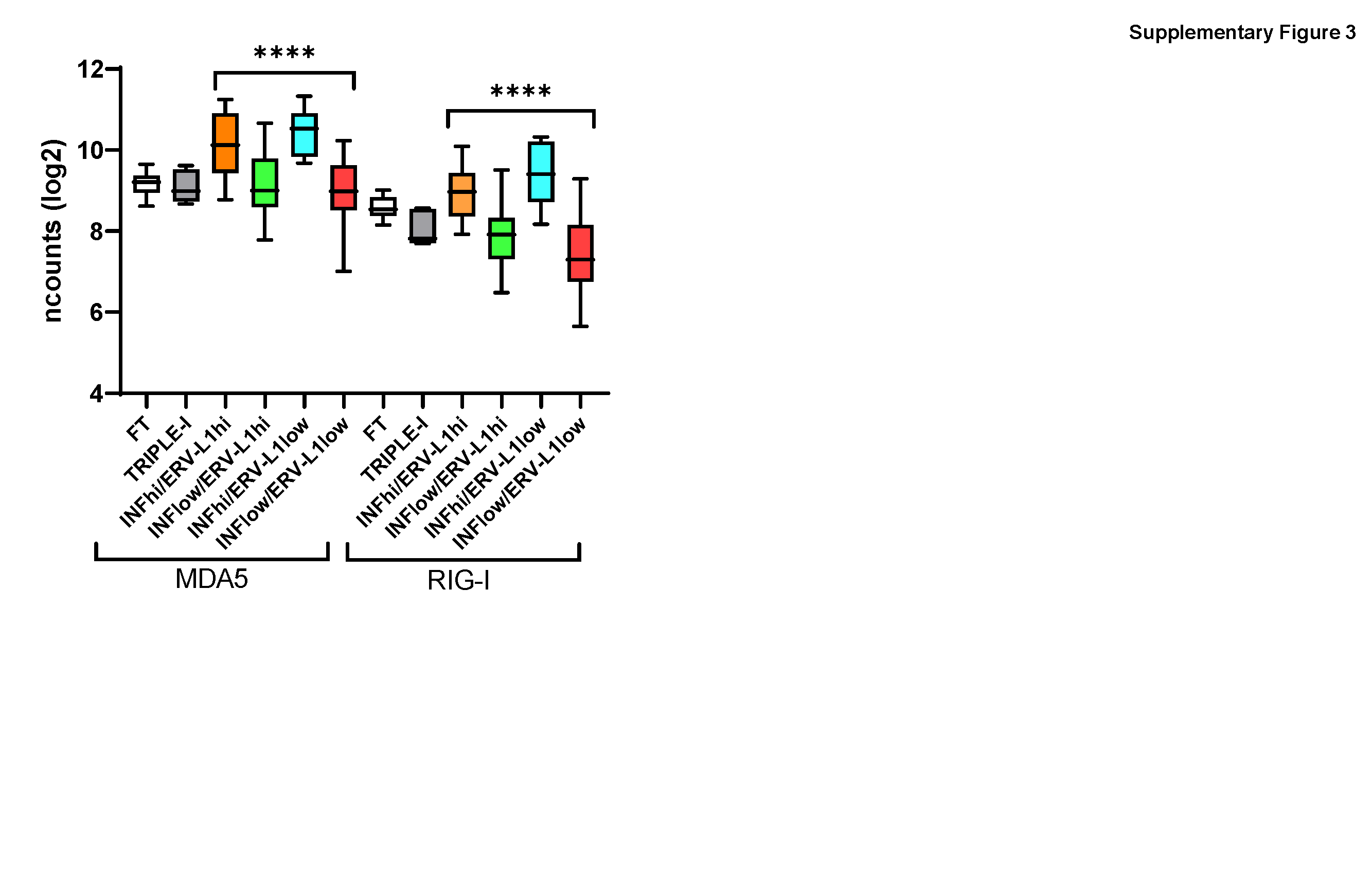

Supplement: Supplementary file 3 — Fig. S3. DsRNA sensors can be deregulated blocking inflammation. [file MOL2-19-3750-s006.tiff]

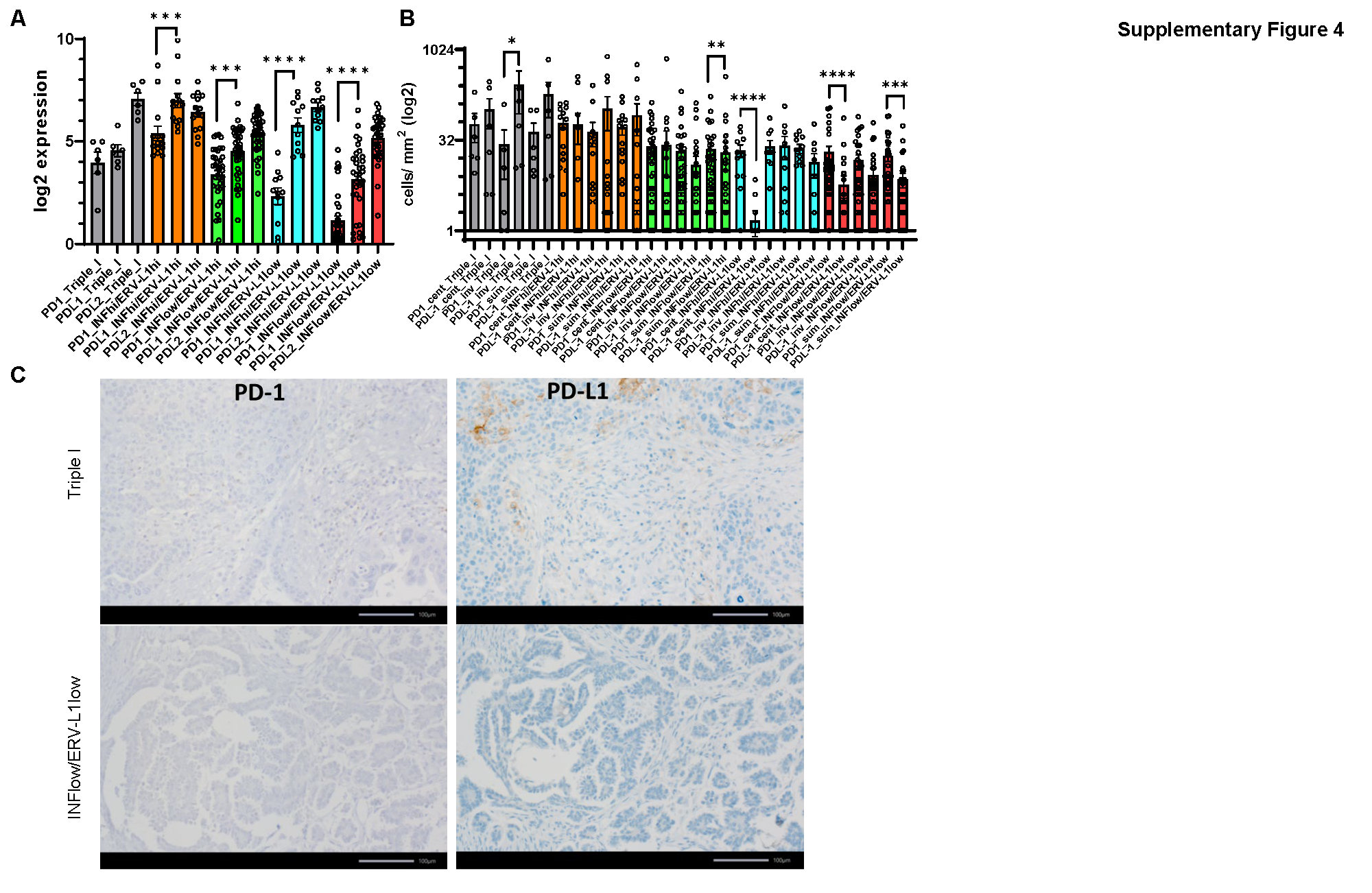

Supplement: Supplementary file 4 — Fig. S4. Immune Checkpoint regulators PD1, PD‐L1, PD‐L2 specifically associate with immune cells of HGSOC. [file MOL2-19-3750-s004.tiff]

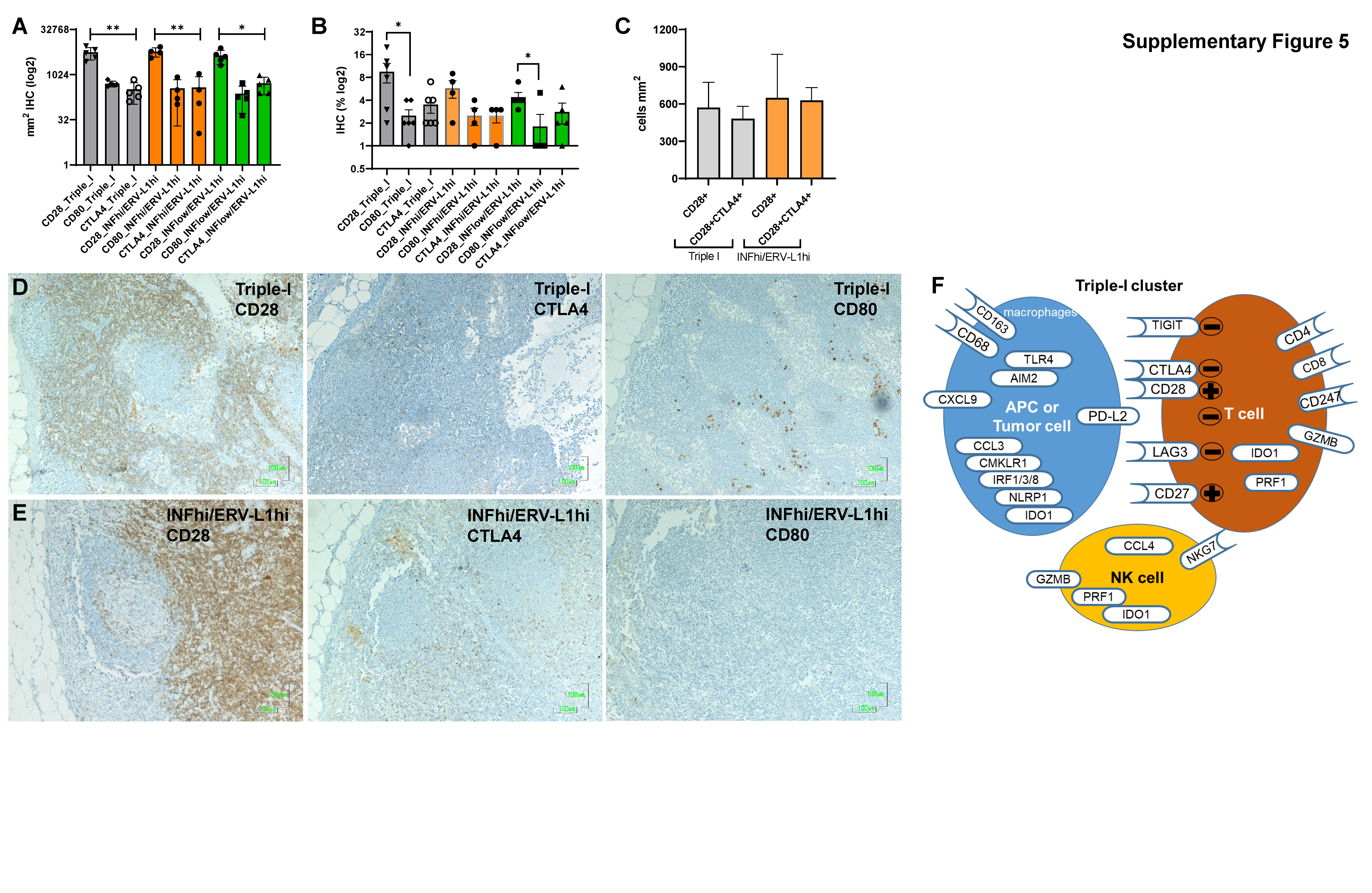

Supplement: Supplementary file 5 — Fig. S5. Comparison of protein expression of immune checkpoint regulators (CD28, CTLA4, CD80) between lymph nodes and the HGSOC TIME from the same patients using IHC. [file MOL2-19-3750-s003.tiff]
